# Ulceration of the Rectum

**Published:** 1845-09

**Authors:** 


*Ulceration of the Rectum.*—James M., aged 40, a laborer, tall, and appears to have been robust, though he has now a somewhat unhealthy aspect, from a slightly icteroid condition of the conjunctiva and skin. He was formerly a sailor, and besides making several voyages to the West Indies, at one period resided there for eight years; he was then very intemperate in his habits, and had an attack of hepatic disease, with jaundice. He was admitted, in the first instance, under the care of the physician, but during his residence he began to be troubled with pain upon passing his stools; this increased, and he states it almost prevented him from passing them at all; when he did so, it was accompanied with considerable sharp pain, which continued half an hour after; he frequently observed a few drops of blood upon his linen after the evacuation. He consequently came under Mr. Luke, and the rectum being inspected by means of a speculum, a superficial ulcer, about the size of a threepenny piece, was discovered at its posterior part, just within the sphincter. The sphincter was divided by cutting directly through the ulcer. The next time he evacuated the bowels, he had lost the pain previously felt, and experienced a mere soreness from the incision. To continue the sulphur electuary which he used previously to the operation. In ten days he had quite lost all the former unpleasant symptoms, and left the surgical wards.

*Remarks.*—This is an example of a class of cases of exceedingly practical importance, in consequence of the great suffering they give rise to, and the fact of their being not unfrequently overlooked. They are also interesting from the marked and speedy relief afforded by the division of the sphincter, in whatever way it may act.—*Ibid.*

